# Effectiveness of nonpharmacological therapeutic interventions on pain and physical function in adults with rib fractures during acute care: A systematic review and meta-analysis

**DOI:** 10.4102/sajp.v78i1.1764

**Published:** 2022-06-28

**Authors:** Beverley J. Weinberg, Ronel Roos, Heleen van Aswegen

**Affiliations:** 1 Department of Physiotherapy, Faculty of Health Sciences, University of the Witwatersrand, Johannesburg, South Africa; 2 The Wits – JBI Centre for Evidenced-Based Practice: A Joanna Briggs Institute Affiliated Group, Johannesburg, South Africa

**Keywords:** acute care, chest trauma, nonpharmacological therapeutic interventions, pain, pneumonia, rib fractures, physical function, rehabilitation

## Abstract

**Background:**

Rib fractures are a common thoracic injury and notable source of chest pain. Chest pain may lead to compromised respiratory and physical function.

**Objectives:**

Our study aimed to synthesise the evidence on the effectiveness of nonpharmacological therapeutic interventions on pain and physical function in adults admitted with rib fractures to acute care settings. Secondary outcomes included length of stay (LOS), respiratory complications, respiratory function and mortality rate.

**Method:**

A systematic literature search of English articles in nine databases was conducted. The Joanna Briggs Institute’s System for the Unified Management, Assessment and Review of Information (SUMARI) was used to conduct our study. Articles written from January 2000 to December 2017 were considered and a search update was completed in 2021. Meta-analysis was conducted for pre- versus post-bundle of care implementation for LOS, pneumonia incidence and mortality rate. Certainty of evidence was appraised using the grading of recommendations, assessment, development and evaluation (GRADE) approach.

**Results:**

Sixteen studies were included (*n* = 2034). Certain interventions were shown to improve respiratory function and reduce pain, pulmonary complications, LOS and mortality rate. No interventions were identified which objectively improved physical function. Meta-analysis showed a statistically significant reduction in relative risk of developing pneumonia (*p* = 0.00) by 63% following bundled care implementation. Certainty of evidence for this outcome was rated as very low following GRADE appraisal.

**Conclusion:**

Nonpharmacological therapeutic interventions used in combination with pharmacological management are viable treatment options to reduce pain, improve respiratory function and reduce the incidence of respiratory complications following acute rib fractures.

**Clinical implications:**

Acupuncture, transcutaneous electrical nerve stimulation (TENS), noninvasive ventilation (NIV) modalities, physiotherapy techniques and multidisciplinary pathways used alongside pharmacological interventions are effective modalities for use in the treatment of acute rib fractures. Multidisciplinary care pathways are important management strategies and reduce the risk of developing pneumonia.

## Introduction

Trauma is a leading cause of death and disability worldwide, with chest trauma directly accounting for over 20% of these deaths (Zimmerman et al. 2020). Of all trauma admissions to emergency departments, more than 15% occur as a result of blunt chest trauma, with rib fractures occurring in 60% – 80% of patients (Dogrul et al. 2020). Rib fractures are a notable source of chest pain and are important indicators of injury severity (Witt & Bulger 2017). Chest pain on inspiration is the main presenting symptom following injury, adversely affecting ventilation (Dogrul et al. 2020). Inability to breathe deeply, cough and clear secretions effectively results (Kim & Moore 2020). Pulmonary complication risk rises, hospital length of stay (LOS) and costs of care increase (Ekpe & Eyo 2016; Farley et al. 2020; Pharaon, Marasco & Mayberry 2015).

Movement of the thorax exacerbates chest pain following rib fractures, restricting physical function (Kerr-Valentic et al. 2003). Disrupted sleep, limitation of bed mobility and mobilisation occur (Bilalee, Maneewat & Sae-Sia 2017; Ho et al. 2014; Kerr-Valentic et al. 2003; Kim & Moore 2020), increasing the risk of immobility-associated complications (Saunders 2015). Unmanaged initial pain may also lead to chronic pain symptoms, reduced quality of life (QOL) and long-term disability (Fabricant et al. 2013; Kerr-Valentic et al. 2003; Marasco et al. 2015; Pharaon et al. 2015).

To avoid complications and improve patient compliance and tolerance to treatment interventions, early effective pain management is essential (Dogrul et al. 2020; Kim & Moore 2020). Pain control options are varied and include pharmacological and nonpharmacological alternatives (Farley et al. 2020). Nonpharmacological therapeutic interventions refer to all rehabilitative and treatment modalities and interventions utilised by healthcare professionals in the acute care setting in the management of pain and rehabilitation of function following acute rib fractures (Weinberg 2020). Using these interventions negates potential adverse side effects associated with pharmacological and operative management, thus benefiting vulnerable populations (Curtis et al. 2016).

No concise summary currently exists in the literature which identifies and evaluates the effects of nonpharmacological therapeutic intervention use in the acute rib fracture population. Our systematic review aimed to identify nonpharmacological interventions which can be utilised to treat pain and rehabilitate function following acute rib fractures in adults and determine the effects of these interventions on pain and physical function. Secondary outcomes included effects on hospital LOS, respiratory function, respiratory complications and mortality rate, hospital re-admission rates because of blunt thoracic chest trauma pain and outcomes related to QOL following traumatic blunt thoracic chest trauma.

## Method

Our systematic review was performed and reported in adherence to the Preferred Reporting Items for Systematic Reviews and Meta-Analyses (PRISMA) guidelines (Moher et al. 2009).

The review protocol was registered in the International Prospective Register of Systematic Reviews (PROSPERO) (reference number CRD42018089060). The protocol was published in the JBI Database of Systematic Reviews and Implementation Reports, reference number: JBISRIR-2017-003600.

Published studies written in or translated into English from 2000 until December 2017 were eligible for inclusion. This search timeframe was used as early trauma management and treatment has changed and advanced significantly over the last 20 years (Unsworth, Curtis & Asha 2015). An updated search was conducted in PubMed for the period 01 January 2018 to 19 January 2021. Randomised controlled trials (RCTs) and observational study designs were considered. Systematic review, research syntheses and text and opinion studies were included for narrative synthesis to report on current best evidence.

The inclusion criteria for studies were as follows:

1. Patients 18 years or older were included who could describe and evaluate their pain, and who were admitted to an acute care setting (intensive care unit [ICU], high care unit or ward) with radiologically confirmed rib fractures sustained via blunt chest trauma.
2. Nonpharmacological therapeutic interventions (standalone or in addition to pharmacological management) were implemented to treat pain or rehabilitate physical function.
3. The primary and secondary outcomes were evaluated, and how these outcomes were assessed was documented.

The exclusion criteria encompassed:

1. Patients who sustained penetrating chest wall trauma
2. Patients who possessed comorbidities preventing physical activity or mobilisation (acute spinal cord injury, polytrauma [complex lower limb and orthopaedic injuries] or traumatic brain injuries)
3. Patients who were pregnant, had psychiatric illness or had abdominal or cranial surgeries
4. Patients whose primary or secondary outcomes were not evaluated.

Databases utilised included: MEDLINE using PubMed, Scopus, CINAHL Plus, PsycINFO and PEDro. Google Scholar, OpenGrey (SIGLE), Cochrane Library and the international prospective register of systematic reviews (PROSPERO) were also reviewed. Reference lists of included studies and relevant journal articles were scanned for additional studies.

### Search strategy

The PubMed Medical Subject Headings (MeSHs) database was utilised to establish all relevant terms. Main concepts searched included rib fractures, pain, physical function and respiratory function. The PubMed search syntax can be viewed in Online Appendix 1. The MeSH terms and search combinations and strategies utilised for ‘setting’ and ‘nonpharmacological therapeutic interventions’ are recorded in Online Appendix 1. The first and second authors independently screened all titles and abstracts of retrieved records to identify studies meeting eligibility criteria for full-text review. Reviewers evaluated full-text articles retrieved and made a final selection of relevant studies. Disagreements were resolved through discussion and consensus or with the third author. Relevant studies were independently evaluated for methodological validity prior to inclusion.

### Data collection process

The first two authors independently extracted data from included articles. To ensure quality assurance, reviewers extracted data from the first five articles individually and thereafter met to determine if their method of data extraction was consistent with the review question before continuing separately. Data extracted included details regarding the populations, study methods, interventions implemented and outcomes of significance to the review question. The first author contacted manuscript authors for clarification or additional information where data were incomplete.

### Risk of bias

Risk of bias was evaluated at both a study and outcome level. Studies were independently appraised for methodological quality and risk of bias utilising the JBI standardised critical appraisal tools. Reviewers met to compare appraisal scores following independent assessment and any discrepancies were resolved via discussion. Critical appraisal scores per study are recorded in Online Appendix 1. At outcome level, the grading of recommendations, assessment, development and evaluation (GRADE) approach for rating the certainty of evidence was utilised (GRADEpro GDT 2015). Following criteria evaluation, certainty of evidence ratings were generated in the summary of findings table. The summary of findings table can be viewed in the Online Appendix 1.

### Summary measures

For dichotomous data, relative risk was utilised as the effect measure and a 95% confidence interval was set. Mean difference was utilised for continuous data.

### Synthesis of results

Results were pooled into a statistical meta-analysis utilising the JBI-SUMARI software programme when at least two studies were similar in the population studied, methodologies utilised, intervention of interest and outcomes measured. Meta-analysis was conducted for pre- versus post-bundle of care implementation for hospital LOS, incidence of pneumonia and mortality rate. All results were subject to double data entry. Findings are presented narratively where pooling of results was not possible. The fixed effects model was utilised for continuous outcome data (hospital LOS) and the random effects model was utilised for dichotomous data (incidence of pneumonia and mortality rate) as per recommendations relating to model selection (Huedo-Medina et al. 2006; Tufanaru et al. 2015). An inverse variance statistical method was utilised for continuous and dichotomous data.

Heterogeneity was assessed using the *I*^2^ statistic and visual inspection of the forest plots. The *I*^2^ value was also reviewed in conjunction with Tau^2^ in a random effects model analysis (Huedo-Medina et al. 2006). Interpretations of *I*^2^ are based on the proposed explanations by Higgins et al. (2003) and Deeks, Higgins and Altman (2008) as cited in the Joanna Briggs Reviewers manual (wiki.joannabriggs.org 2019), with 0% – 30% representing low heterogeneity, 31% – 50% representing moderate heterogeneity, 51% – 74% representing substantial heterogeneity and 75% – 100% representing considerable heterogeneity.

### Ethical considerations

An ethical clearance waiver was obtained from the Human Research Ethics (Medical) Committee of the University of the Witwatersrand (reference number: W-CJ-170419-1).

## Results

### Study selection

**FIGURE 1:**
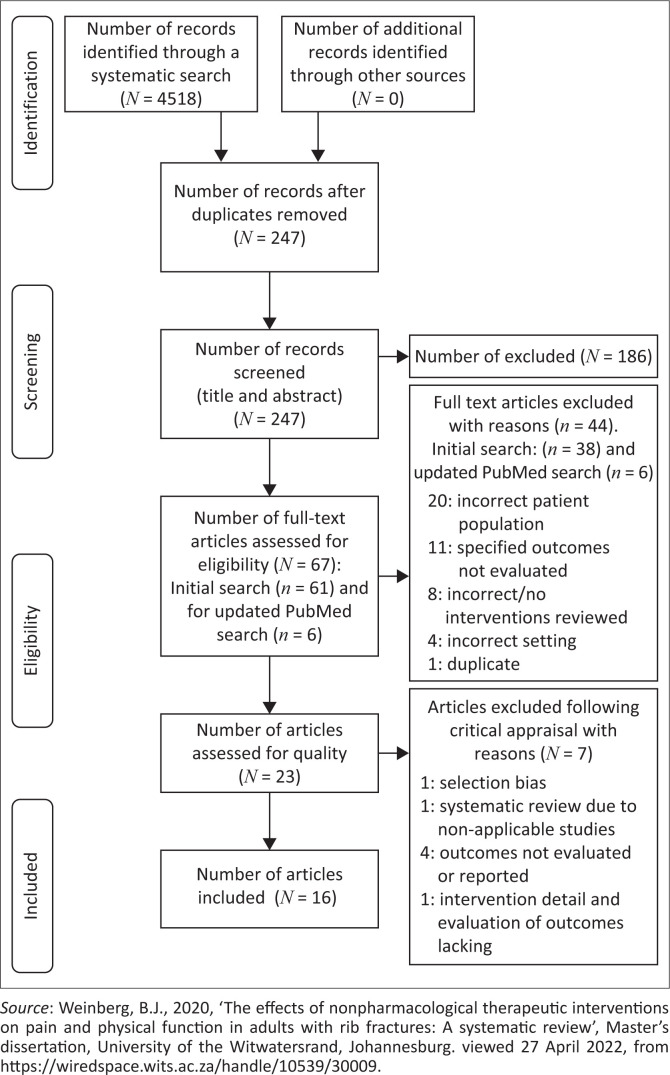
Preferred reporting items for systematic reviews and meta-analyses flow diagram of search and study selection process.

Figure 1 presents the PRISMA flow diagram that outlines the search and study selection process. The initial systematic search yielded 4518 articles (Figure 1). Sixteen articles (Curtis et al. 2016; Easter 2001; Ekpe & Eyo 2016; Flarity et al. 2017; Garfield & Howard-Griffin 2000; Grammatopoulou et al. 2010; Gunduz et al. 2005; Ho et al. 2014; Linton & Sviri 2006; Mehta 2013; Papadopoulos et al. 2017; Sahr et al. 2013; Simon et al. 2012; Todd et al. 2006; Unsworth et al. 2015; Witt & Bulger 2017) from the initial search were selected for final inclusion and carried through for data extraction and synthesis.

### Study characteristics

Our systematic review included three RCTs (Grammatopoulou et al. 2010; Gunduz et al. 2005; Ho et al. 2014), three case reports (Garfield & Howard-Griffin 2000; Linton & Sviri 2006; Papadopoulos et al. 2017), one analytical cross-sectional study (Mehta 2013), four cohort (Curtis et al. 2016; Flarity et al. 2017; Sahr et al. 2013; Todd et al. 2006) and five text and opinion studies (Easter 2001; Ekpe & Eyo 2016; Simon et al. 2012; Unsworth et al. 2015; Witt & Bulger 2017). Studies originated from the following countries: United States of America (USA) (38%) (Easter 2001; Flarity et al. 2017; Sahr et al. 2013; Simon et al. 2012; Todd et al. 2006; Witt & Bulger 2017), Australia (13%) (Curtis et al. 2016; Unsworth et al. 2015), Greece (13%) (Grammatopoulou et al. 2010; Papadopoulos et al. 2017), England (6%) (Garfield & Howard-Griffin 2000), Israel (6%) (Linton & Sviri 2006), India (6%) (Mehta 2013), Nigeria (6%) (Ekpe & Eyo 2016), Taiwan (6%) (Ho et al. 2014) and Turkey (6%) (Gunduz et al. 2005).

Our systematic review sample encompassed *n* = 2034 study participants, with *n* = 864 (42.5%) female participants and *n* = 1180 (58.0%) male participants. Baseline characteristics and clinical profiles of included studies are presented in Online Appendix 1.

Patients’ average ages fell mainly within the category of 50–60 years of age (Flarity et al. 2017; Grammatopoulou et al. 2010; Ho et al. 2014; Todd et al. 2006), followed by those over 65 years (Curtis et al. 2016; Linton & Sviri 2006; Sahr et al. 2013) and those under 50 years (Gunduz et al. 2005; Garfield & Howard-Griffin 2000; Ho et al. 2014). The commonest mechanisms of injury included road traffic or motor vehicle accidents (Garfield & Howard-Griffin 2000; Ho et al. 2014, Papadopoulos et al. 2017) and falls in the elderly (Curtis et al. 2016; Sahr et al. 2013). Multiple rib fractures were reviewed in most studies (Flarity et al. 2017; Grammatopoulou et al. 2010; Gunduz et al. 2005; Ho et al. 2014; Mehta 2013; Sahr et al. 2013; Todd et al. 2006). Age (Curtis et al. 2016; Sahr et al. 2013; Todd et al. 2006), injury severity score (ISS) (Curtis et al. 2016; Flarity et al. 2017; Ho et al. 2014; Sahr et al. 2013; Todd et al. 2006) and number of rib fractures (Curtis et al. 2016; Flarity et al. 2017; Todd et al. 2006) were the most frequently identified variables potentially influencing management and/or outcomes.

Study outcomes reviewed, methods of assessment and interventions implemented varied across included studies. Outcomes evaluated and respective study results are presented in the Online Appendix 1. Table 1 is an outline of the treatment approaches included in sourced studies, which addressed the systematic review outcomes.

**TABLE 1:** Treatment approaches included in studies addressing systematic review outcomes.

Interventions	Pain relief	Improved pulmonary function	Reduced pulmonary complications	Improved physical function	Reduced HLOS	Reduced ICU LOS	Reduced mortality rate
**Acupuncture:**							
Filiform needles	√	√	√	√	-	-	-
Auricular	√	√	-	*Subjective report*	-	-	-
Bundled care/MDT clinical pathways	-	-	√√	-	√√	√√√	√
**Respiratory modalities:**							
ACBT	√	-	√	-	-	-	-
Combination Rx: Conservative management including positioning, early mobilisation, supported coughing and incentive spirometry	√	-	√	-	-	-	-
TENS	√	√	-	-	-	-	-
**Noninvasive ventilation:**							
CPAP (facemask)	√	-	√	-	√	√	√
CNEP	-	√	√	-	-	-	-
CPAP (facemask) and IPPB	-	-	√	-	-	-	-

*Source*: Weinberg, B.J., 2020, ‘The effects of nonpharmacological therapeutic interventions on pain and physical function in adults with rib fractures: A systematic review’, Master’s dissertation, University of the Witwatersrand, Johannesburg. viewed 27 April 2022, from https://wiredspace.wits.ac.za/handle/10539/30009.

ACBT, active cycle of breathing technique; CPAP, continuous positive pressure ventilation; CNEP, continuous negative extrathoracic pressure; HLOS, hospital length of stay; ICU, intensive care unit; IPPB, intermittent positive pressure breathing; LOS, length of stay; MDT, multidisciplinary team; NIV, noninvasive ventilation; Rx, treatment; TENS, transcutaneous electrical nerve stimulation.

√: Number of studies documenting a positive outcome.

Bundled care or MDT clinical pathway includes and organises transdisciplinary interventions as a means of optimising compliance to recommended treatment guidelines (Unsworth et al. 2015). Bundled care and MDT clinical pathways had the highest number of studies addressing specific outcomes of the systematic review.

Bundled care and clinical pathway implementation were evaluated in more than one study relative to the outcomes of hospital LOS (Curtis et al. 2016; Flarity et al. 2017; Todd et al. 2006), ICU LOS (Flarity et al. 2017; Sahr et al. 2013; Todd et al. 2006), mortality rate (Curtis et al. 2016; Flarity et al. 2017; Todd et al. 2006) and pneumonia incidence (Curtis et al. 2016; Todd et al. 2006).

#### Outcome: Hospital length of stay

Four studies (Curtis et al. 2016; Flarity et al. 2017; Sahr et al. 2013; Todd et al. 2006) reported on the outcome of hospital LOS following bundled care implementation. Curtis et al. (2016) implemented a blunt chest injury early activation protocol (CHiP) in an elderly population. The protocol included physiotherapy, pain and trauma team review, patient-controlled analgesia (PCA) and high-flow nasal prong oxygen (HFNP). Todd et al. (2006) implemented an MDT clinical pathway focused on respiratory therapy, pain management, physiotherapy and optimising nutrition services. Respiratory therapy constituted use of nebulisation therapies, EzPAP positive airway pressure system and other escalating invasive therapies as required. Pain control was maximised under the pain service (oral pain medication, NSAIDs, intravenous pain medications and epidural analgesia as deemed appropriate). Physiotherapists involved with the physical rehabilitation of patients aimed to optimise mobility utilising strengthening, range of motion and balance exercises. The MDT care also included nutritional management (Weinberg 2020). Flarity et al. (2017) implemented a pathway to determine the level of patient care needed. It consisted of early bedside forced vital capacity (FVC) assessment, early analgesia and early evaluation of patients who might show respiratory compromise. Pulmonary secretion clearance techniques formed part of this pathway dependent on FVC assessment findings. Sahr et al. (2013) implemented a care pathway that included aggressive pain control including PCA, early mobilisation and multidisciplinary care (physical and occupational therapy, physical medicine, social work and pharmacy).

Three studies (Curtis et al. 2016; Sahr et al. 2013; Todd et al. 2006) provided sufficient information for inclusion in a meta-analysis and are reviewed below the respective forest plot. Flarity et al. (2017) was excluded from the meta-analysis (necessary data format requested was not supplied) and demonstrated no difference in LOS (*p* = 0.095) in the total study cohort following bundled care intervention. Sahr et al. (2013) recorded two data sets relative to the number of rib fractures (greater or less than three rib fractures) and impact on hospital LOS following pathway implementation in elderly patients. A statistically significant decrease in LOS favouring post protocol implementation (*p* = 0.006) was shown. Curtis et al. (2016) demonstrated a nonsignificant difference (*p* = 0.50) between cohorts for hospital LOS following implementation of a blunt chest injury early activation protocol (ChIP). Following adjustment for confounding variables, this nonsignificant difference remained (adjusted mean difference in LOS −0.2 days, 95% CI −1.2 to 0.8, *p* = 0.74). The authors speculated that the higher median ISS found in the post-ChIP cohort may explain the lack of effect of pathway implementation on LOS. It was found that LOS (especially in elderly populations) is affected by many factors, not only the management implemented. Todd et al. (2006) demonstrated that in patients 45 years and older with greater than four rib fractures, post-pathway patients had nonsignificantly decreased hospital LOS, 14.3 (+/−16.9) days in the pre- versus post-pathway cohort 11.7 (± 10.9) with *p* = 0.11 on unadjusted univariate analysis.

#### Outcome: Intensive care unit length of stay

Four studies reported on the outcome of ICU LOS (Flarity et al. 2017; Gunduz et al. 2005; Sahr et al. 2013; Todd et al. 2006), with three (Flarity et al. 2017; Sahr et al. 2013; Todd et al. 2006) reviewing bundled care intervention relative to ICU LOS. As a result of necessary data not being supplied, only two studies (Sahr et al. 2013; Todd et al. 2006) were included in the meta-analysis. The meta-analysis however revealed high heterogeneity (*I*^2^ = 63% with random effects model), making pooling of results inappropriate. Individual study results are reviewed narratively. Flarity et al. (2017) documented a reduction in ICU LOS by over 2 days in the postclinical practice guideline (CPG) ICU cohort, despite this cohort being significantly older with more rib fractures. Todd et al. (2006) showed a decreased ICU LOS by 2.4 days (*p* = 0.01) following MDT pathway implementation in patients older than 45 years of age with more than four rib fractures. Whilst Sahr et al. (2013) found that patients with more than three rib fractures had a longer ICU LOS (as a result of injury severity) compared with patients with fewer than three rib fractures, even following protocol intervention.

#### Outcome: Incidence of pneumonia

Three studies (Curtis et al. 2016; Gunduz et al. 2005; Todd et al. 2006) reported on the outcome of pneumonia incidence. Two studies reviewed pneumonia incidence following bundled care implementation (Curtis et al. 2016; Todd et al. 2006), and meta-analysis was conducted. Curtis et al. (2016) documented a 4.8% reduction in pneumonia incidence (95% CI 0.5–9.2, *p* = 0.03) following ChIP implementation in elderly patients. This difference remained significant following adjustment for confounding variables, with the odds of developing pneumonia being 56% lower in the after-ChIP cohort (OR 0.44, 95% CI 0.21–0.90, *p* = 0.03). Todd et al. (2006) showed decreased pneumonia incidence following pathway implementation on univariate (*p* = 0.0003) and multivariant analyses (OR 0.12, 95% CI 0.04–0.34, *p* < 0.001) in patients 45 years and older with more than four rib fractures.

Pulmonary morbidity was also found to be reduced following respiratory screening protocol use (Flarity et al. 2017; Todd et al. 2006; Witt & Bulger 2017), allowing for early detection of patients at high risk of respiratory compromise, and prompt directed management to be implemented.

#### Outcome: Mortality rate

Mortality rate was reported as an outcome in four of the included studies (Curtis et al. 2016; Flarity et al. 2017; Gunduz et al. 2005; Todd et al. 2006). Three that reviewed bundled care intervention relative to its effect on mortality rate were included in the meta-analysis (Curtis et al. 2016; Flarity et al. 2017; Todd et al. 2006). Curtis et al. (2016) documented no significant difference in mortality rate between pre- and post-ChIP cohorts (95% CI −0.8 to 4.0, *p* = 0.29) following bundled care implementation in an elderly population. Whilst Todd et al. (2006) reported a reduction in mortality rate in patients 45 years and older with more than four rib fractures following clinical pathway intervention, on both unadjusted univariate analysis (*p* = 0.004) and following adjustment for age, ISS and number of rib fractures (OR 0.37, 95% CI 0.13–1.03, *p* = 0.06). Flarity et al. (2017) reviewed mortality rate relative to two patient populations (hospital admission and ICU admission) following pre- and post-CPG implementation. Each population had their own mortality analysis, as presented in the forest plot. Pre- and post-CPG cohorts demonstrated no difference in mortality rate between groups (*p* = 0.763) or between pre- and post-CPG ICU cohorts (*p* = 0.951) (Flarity et al. 2017), indicating no difference in the hospital or ICU populations following CPG intervention.

#### Outcome: Pain

Pain was reviewed as an outcome in five (Grammatopoulou et al. 2010; Gunduz et al. 2005; Ho et al. 2014; Mehta 2013; Papadopoulos et al. 2017) of the final studies. Methods of pain evaluation and interventions implemented to influence pain, however, varied across studies. As a result of this diversity, pooling of results was not possible. Findings are presented in Online Appendix 1. Pain-relieving interventions identified included: TENS (Mehta 2013), filiform acupuncture (Ho et al. 2014), auricular acupuncture intervention (Papadopoulos et al. 2017), ACBT (Grammatopoulou et al. 2010) and NIV modalities (Gunduz et al. 2005).

#### Outcome: Physical function

Only subjective improvement in physical function was observed by Ho et al. (2014) following filiform acupuncture intervention. No studies included in this review evaluated physical function via objective outcome measures.

#### Outcome measure: Respiratory function

Four of the included studies (Grammatopoulou et al. 2010; Ho et al. 2014; Mehta 2013; Papadopoulos et al. 2017) reviewed respiratory function as an outcome using differing methods and measures of evaluation following implementation of TENS (Mehta 2013), filiform acupuncture (Ho et al. 2014), physiotherapy intervention (Grammatopoulou et al. 2010) and auricular acupuncture (Papadopoulos et al. 2017). Pooling of results was not possible because of this diversity. Study results are reviewed in Online Appendix 1. Use of respiratory screening measures (Flarity et al. 2017; Todd et al. 2006; Witt & Bulger 2017) was found to positively influence respiratory function, allowing for early detection of respiratory compromise and timely management to be implemented.

### Meta-analysis results

#### Pre- versus post-bundle of care implementation on hospital length of stay

**FIGURE 2:**
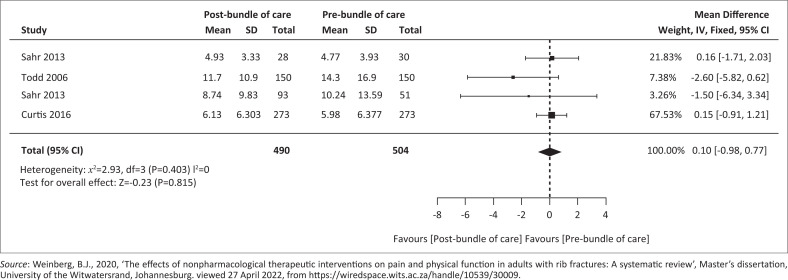
Forest plot of pre- versus post bundle of care implementation on hospital length of stay.

Figure 2 outlines the pre- versus post-bundle of care implementation on hospital LOS. Pooled analysis of three cohort studies (Curtis et al. 2016; Sahr et al. 2013; Todd et al. 2006) with 994 participants (*n* = 490 post-bundle of care; *n* = 504 pre-bundle of care) demonstrated a mean difference of −0.10 (95% CI −0.98 to 0.77) following bundled care intervention, with *p* = 0.82 and *I*^2^= 0% (Weinberg 2020).

#### Pre- versus post-bundle of care implementation on pneumonia incidence

**FIGURE 3:**
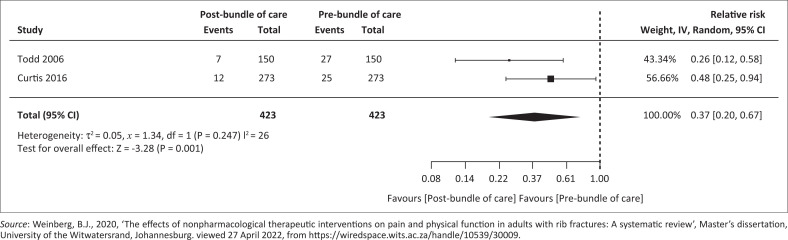
Forest plot of pre- versus post-bundle of care implementation on pneumonia incidence.

Figure 3 outlines the pre- versus post-bundle of care implementation on pneumonia incidence. Pooled analysis of two cohort studies (Curtis et al. 2016; Todd et al. 2006) yielded 846 participants (*n* = 423 per group). Results revealed a RR of 0.37 (95% CI 0.20–0.67) with *p* = 0.00. Patients managed with care bundles had a 63% reduction in RR of developing pneumonia compared with those managed without care bundles. Tau^2^ = 0.05 and *I*^2^ =26% (Weinberg 2020).

#### Pre- versus post-bundle of care implementation on mortality rate

**FIGURE 4:**
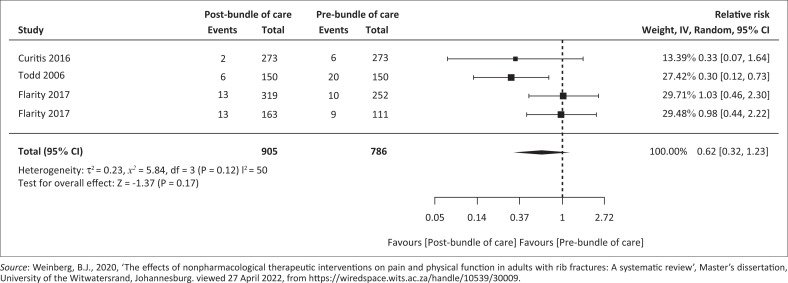
Forest plot of pre- versus post-bundle of care implementation on mortality rate.

Figure 4 presents the forest plot related to pre- versus post-bundle of care implementation on mortality rate. Pooled analysis of three cohort studies (Curtis et al. 2016; Flarity et al. 2017; Todd et al. 2006) included 1691 participants (*n* = 905 post-bundle of care; *n* = 786 pre-bundle of care). Meta-analysis demonstrated a RR of 0.62 (95% CI 0.32–1.23) with *p* = 0.12 and *I*^2^ = 50% and Tau^2^ = 0.23. Mortality risk showed no significant reduction following bundled care implementation (Weinberg 2020).

## Discussion

Findings of our systematic review show that implementation of nonpharmacological therapeutic interventions in combination with pharmacological management decrease pain and reduce the incidence of pulmonary complications following acute rib fractures. Improvements in respiratory function and influences on the outcomes of LOS and mortality rate were also demonstrated. Notably limited studies reviewing nonpharmacological therapeutic interventions relative to the set outcomes were retrieved in the literature, alongside use of differing assessment methods relative to the interventions implemented. Main findings from our review do, however, show that acupuncture intervention, continuous positive airway pressure (CPAP) via facemask, physiotherapy modalities (ACBT, supported cough, incentive spirometry, intermittent positive pressure breathing, optimal positioning, early mobilisation) and transcutaneous electrical nerve stimulation (TENS) therapy are nonpharmacological therapeutic interventions which relieve pain following rib fractures. Subjective improvements in physical function were observed following acupuncture implementation.

Acupuncture, TENS and noninvasive ventilation (NIV) modality use improved respiratory function, whilst respiratory screening protocols assisted in reducing pulmonary morbidity. Utilisation of ACBT, acupuncture therapy, NIV and MDT clinical pathways reduced pulmonary complications. Interventions resulting in reduced LOS and mortality rate included NIV modalities and MDT clinical pathways and care bundles.

Multidisciplinary care and clinical pathways were identified as essential treatment practices in the management of patients following acute rib fractures (Curtis et al. 2016; Flarity et al. 2017; Sahr et al. 2013; Todd et al. 2006; Unsworth et al. 2015; Witt & Bulger 2017).

Aggressive pain intervention, early mobilisation, multidisciplinary care, physiotherapy or respiratory therapy, including pulmonary toilet and ventilatory support, were modalities included in bundled care pathways. Upright positioning for improved ventilation and physical comfort, incentive spirometry and family and patient education were also implemented in some care bundles (Easter 2001; Unsworth et al. 2015; Witt & Bulger 2017). Pathway implementation resulted in standardisation of practice and facilitated multidisciplinary involvement. Use of care bundles holds potential to significantly reduce pneumonia incidence following rib fractures, and acute care facilities should consider integrating these pathways into patient care.

The outcomes of hospital LOS and mortality rate showed nonstatistically significant differences following bundled care implementation. Certainty of evidence ratings (GRADE) were appraised as moderate for the outcome of hospital LOS and very low for the outcome of mortality rate. These outcomes were reported to be influenced by certain factors (e.g. age), other than the interventions reviewed. Findings concur with previous studies (Bulger et al. 2000; Bergeron et al. 2003), where extended LOS and delays in discharge were reported in the elderly as a result of greater susceptibility to complications (Bergeron et al. 2003). Increased morbidity and mortality risk in the elderly was also demonstrated in comparison to younger patients with similar injuries following rib fractures (Bulger et al. 2000). Dalton et al. (2019) concluded that increased age was the only variable predicting with statistical significance, failure to achieve expedited discharge in those having sustained multiple traumatic rib fractures. Consequently, as LOS in the elderly is influenced by many factors and not only the management implemented, this may account for the overall nonsignificant difference observed for LOS on meta-analysis (which included a predominantly older population) in our review. Furthermore, although results were considered statistically insignificant for this outcome, a possible reduction in LOS by 1 day may be of clinical and financial significance both for the patient and hospital, especially in countries where these resources are limited (Weinberg 2020). In a study exploring the effects of expedited discharge of patients with multiple traumatic rib fractures relative to cost-effectiveness, results demonstrated average costs of hospitalisation for those who achieved expedited discharge were less than half of the average cost for those who did not (Dalton et al. 2019). Additional benefits from rapid discharge for patients included reduced risk of acquiring nosocomial infections and earlier return to their normal activities. These findings support that our deductions, and reductions in LOS, although not of statistical significance, may still provide benefit, decreasing risks associated with prolonged hospital LOS and costs incurred.

The main limitations to our systematic review and meta-analysis are acknowledged. Studies without radiologically diagnosed rib fractures were excluded to standardise inclusion criteria. As rib fractures may however be missed following chest X-ray evaluation (Sano 2018), studies may have inadvertently been omitted. Pooling of results (for the outcomes of pain and respiratory function) were limited because of methods of evaluation, interventions implemented and outcome measures assessed varying considerably amongst studies. In addition, no studies evaluated hospital re-admission rates because of blunt thoracic chest trauma pain or evaluated outcomes related to QOL following this injury. Limitations of meta-analysis included inclusion of a small number of studies, and only cohort studies, which are at higher risk of bias than RCTs (Lavallée et al. 2017).

## Conclusion

Nonpharmacological therapeutic interventions used in conjunction with pharmacological management aided pain relief, improved respiratory function and reduced the incidence of pneumonia following rib fractures in the acute care setting. Bundled care pathways were identified as important management strategies in the treatment of patients, aiding identification of early respiratory compromise and promoting standardisation of care and multidisciplinary team collaboration.
